# Long-term exposure of human endothelial cells to metformin modulates miRNAs and isomiRs

**DOI:** 10.1038/s41598-020-78871-5

**Published:** 2020-12-11

**Authors:** Angelica Giuliani, Eric Londin, Manuela Ferracin, Emanuela Mensà, Francesco Prattichizzo, Deborah Ramini, Fiorella Marcheselli, Rina Recchioni, Maria Rita Rippo, Massimiliano Bonafè, Isidore Rigoutsos, Fabiola Olivieri, Jacopo Sabbatinelli

**Affiliations:** 1 Department of Clinical and Molecular Sciences, Università Politecnica Delle Marche, Via Tronto 10/A, 60126 Ancona, Italy; 2 Computational Medicine Center, Thomas Jefferson University, Philadelphia, PA USA; 3 Department of Experimental, Diagnostic, and Specialty Medicine (DIMES), University of Bologna, Bologna, Italy; 4 IRCCS MultiMedica, Milan, Italy; 5 Center of Clinical Pathology and Innovative Therapy, IRCCS INRCA, Ancona, Italy

**Keywords:** Senescence, miRNAs

## Abstract

Increasing evidence suggest that the glucose-lowering drug metformin exerts a valuable anti-senescence role. The ability of metformin to affect the biogenesis of selected microRNAs (miRNAs) was recently suggested. MicroRNA isoforms (isomiRs) are distinct variations of miRNA sequences, harboring addition or deletion of one or more nucleotides at the 5′ and/or 3′ ends of the canonical miRNA sequence. We performed a comprehensive analysis of miRNA and isomiR profile in human endothelial cells undergoing replicative senescence in presence of metformin. Metformin treatment was associated with the differential expression of 27 miRNAs (including miR-100-5p, -125b-5p, -654-3p, -217 and -216a-3p/5p). IsomiR analysis revealed that almost 40% of the total miRNA pool was composed by non-canonical sequences. Metformin significantly affects the relative abundance of 133 isomiRs, including the non-canonical forms of the aforementioned miRNAs. Pathway enrichment analysis suggested that pathways associated with proliferation and nutrient sensing are modulated by metformin-regulated miRNAs and that some of the regulated isomiRs (e.g. the 5′ miR-217 isomiR) are endowed with alternative seed sequences and share less than half of the predicted targets with the canonical form. Our results show that metformin reshapes the senescence-associated miRNA/isomiR patterns of endothelial cells, thus expanding our insight into the cell senescence molecular machinery.

## Introduction

Metformin is a hypoglycemic drug used as a first-line treatment for newly diagnosed type 2 diabetes patients^1^. Over the years, metformin has been shown to exert a geroprotective action, beyond its primary glucose-lowering effect^2^. In particular, recent metanalyses showed a significantly lower rate of all-cause mortality and age-related disease (ARD) incidence associated with metformin treatment, thus suggesting that this drug may extend lifespan and disease-free survival in diabetic subjects even compared with non-diabetic people^3,4^.

These evidence prompted the launching of the controlled clinical trial Targeting Aging with Metformin (TAME), in order to test whether metformin can delay the onset of ARDs in healthy (non-diabetic) aged subjects^5^. However, although the clinical outcomes of metformin as a pharmacological intervention to achieve healthy longevity are currently being investigated, the exact mechanisms of action remain elusive^6^. At the cellular level, metformin acts on several pathways which are recognized as molecular pillars of cell senescence, including inflammation, autophagy, proteostasis and cellular survival^2,7,8^. While metformin has been shown to influence the cellular epigenetic machinery by modulating Sirtuin-1 (SIRT1)^9^, i.e. the pro-longevity histone deacetylase^10^, few studies have attempted to identify changes in expression profiles of microRNAs (miRNAs, miRs) induced by metformin treatment in the framework of cellular senescence, mainly showing a general increased abundance of multiple miRNAs after a short-term, high-dose treatment^11,12^. Due to their capability of preventing translation of specific messenger RNAs (mRNA), miRNAs, can impact many cellular processes, including cellular senescence^13^. The knowledge of miRNAs has significantly improved with the advent of next-generation sequencing (NGS) technologies. Indeed, small RNA sequencing (small RNA-seq) of miRNAs enables the analysis of the expression of thousands of miRNAs, the concurrent discovery of new miRNAs, and confirmation of known miRNAs^14^. Moreover, bioinformatic analyses of small RNA-seq data have shown that multiple miRNA isoforms, commonly named isomiRs, can be generated from the processing of each precursor miRNA^15^. IsomiRs present with the addition or deletion of one or more nucleotides at the 5′ and/or 3′ ends of the canonical miRNA sequence and are thought to be produced as distinct products rather than being transcription errors^16,17^. Beyond these genetically encoded variants, miRNAs can undergo post-transcriptional sequence modifications resulting in non-template uridylation at the 3′ end^18^. Growing evidence showed that these modifications can affect the stability of the RNA sequence, confer different targets compared the canonical mature form^19^, or affect the subcellular compartmentalization of the miRNA^20^. However, their biological significance is still under discussion.

Based on the evidence that treatment with metformin can modulate in vitro cellular senescence, as well as the biogenesis of miRNAs^12^, we performed for the first time a miR-seq analysis of human umbilical vein endothelial cells (HUVECs) undergoing replicative senescence in the presence of pharmacologically pertinent doses of metformin in order to identify senescence-associated (SA) miRNA and isomiR signatures affected by metformin treatment.

## Results

### Modulation of miRNA patterns induced by metformin treatment in senescent endothelial cells

**Figure 1:**
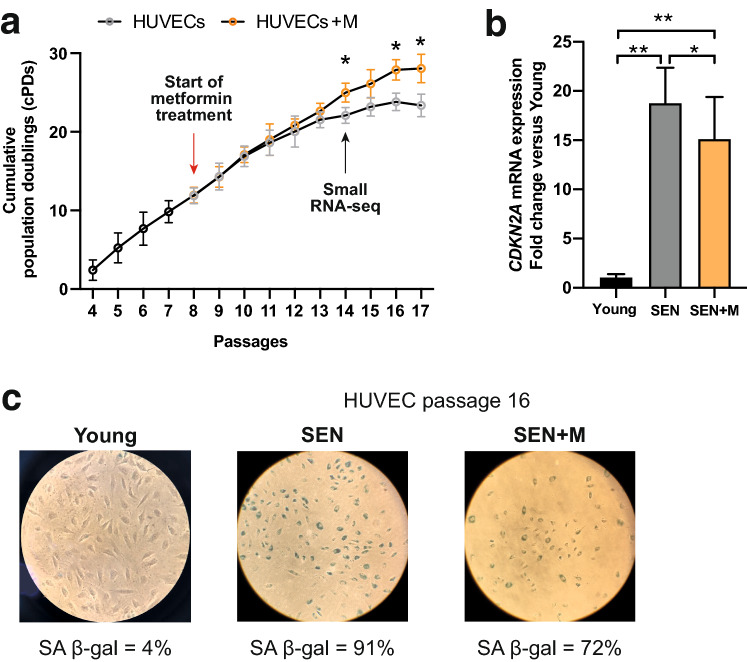
Characterization of replicative senescence in HUVECs. (**a**) Cumulative population doubling (cPD) curves. Metformin treatment was started after passage 7 (cPD = 9.83) and conducted at each medium replacement until complete growth arrest. (**b**) *CDKN2A* mRNA relative expression in young, SEN and SEN + M. Data are mean ± SD. *p < 0.05; **p < 0.01. (**c**) Representative positivity and quantification of the SA β-Gal staining in young, senescent (passage 16, SEN) and SEN HUVECs treated with metformin (SEN + M). *HUVECs* human umbilical vein endothelial cells, *SA* senescence-associated.

To identify the pool of SA miRNAs modulated by metformin, we used a well-established model of human umbilical vein endothelial cells (HUVECs) undergoing replicative senescence^21,22^. When the proportion of SA β-gal positivity exceeded 10% in replicating cells (cPD = 9.83; Fig. 1a), 20 μM metformin was added at each medium replacement, and cells were cultured until complete growth arrest (SA β-gal positive cells > 80%). We selected this concentration since it falls within the range observed in plasma of patients treated with the lowest doses of metformin^6^. At passage 16, after approximately 60 days, senescent cells (SEN) and senescent cells treated with metformin (SEN + M) were harvested to perform small RNA-seq (Fig. 1a). Interestingly, SEN + M showed an increased population doubling rate (Fig. 1a), a reduced SA β-gal activity (Fig. 1b), and a decreased CDKN2A mRNA expression (Fig. 1c) compared to SEN.

**Figure 2:**
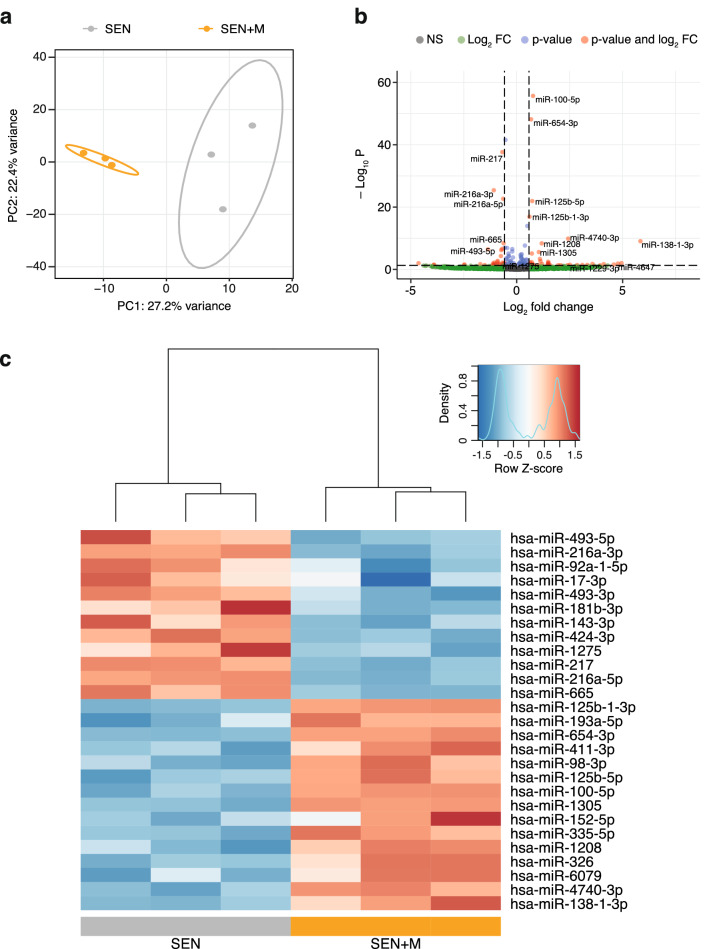
MiR-seq analysis of senescent HUVECs treated with metformin. (**a**) PCA plot of the first two principal components (PC1 and PC2) using transformed normalized miR-seq data. Circles represent 95% confidence intervals. (**b**) Volcano plot of log_2_ fold-changes (FC, SEN + M compared to SEN) vs. -log_10_ adjusted p-values using transformed normalized miR-seq data. MiRNAs with FC ≥ 1.5 (log2 FC ≥ 0.585) and FDR < 0.05 (− log10 p-value < 1.30) are highlighted in red. (**c**) Heatmap showing clustering of samples and miRNAs differentially expressed in SEN + M compared to SEN. Data is shown following Z-score transformation. Red color indicates Z-scores > 0 (above mean), blue colors indicate Z-scores < 0 (below mean). MiRNAs are ranked according to the lowest log_2_ FC.

Pair-wise correlation among normalized reads generated by three biological replicates for each condition gave Pearson correlation coefficients > 0.90 (Supplementary Fig. S1), indicating high correlation among replicates. Normalized miRNA expression data were compared via principal component analysis (PCA). A PCA plot based on principal components 1 and 2, explaining 27.2% and 22.4% of the total variance respectively, showed a clear separation between SEN and SEN + M (Fig. 2a). MiRNAs with a significant moderated *t* test (FDR < 0.05) and an absolute fold change ≥ 1.5 were considered as differentially regulated. The Volcano plot showed log2 fold change and − log10 p-values of all the detected miRNAs (Fig. 2b), while the normalized expressions of differentially expressed miRNAs were displayed in a heatmap (Fig. 2c). Of 1706 miRNAs detected in at least one sample, we identified 27 miRNAs whose expression was altered by metformin. In particular, 15 miRNAs were upregulated and 12 were downregulated in SEN + M (Fig. 2c).

### Changes in the isomiR pattern associated with metformin treatment

Since studying miRNAs at the isomiR level could lend new insights into miRNA biology and function, we analysed isomiR modulation associated with metformin treatment of HUVECs during replicative senescence. IsomiRs result from a shift of the cutting site of Drosha/Dicer enzymatic activities during miRNA biogenesis^23,24^ and can be classified into six categories according to the types of sequence modifications: (1) canonical miRNAs, (2) 3′ deletion isomiRs, (3) 3′ addition isomiRs, (4) 5′ deletion isomiRs, (5) 5′ addition isomiRs, and (6) mixed isomiRs, which represent a combination of the prior categories^25^. We also analysed the post-transcriptional addition of one or more uridines at the 3′ end of isomiRs and canonical miRNAs, namely uridylation. It has to be noted that the entire spectrum of isomiRs is covered by the standard miR-seq analysis.

**Figure 3:**
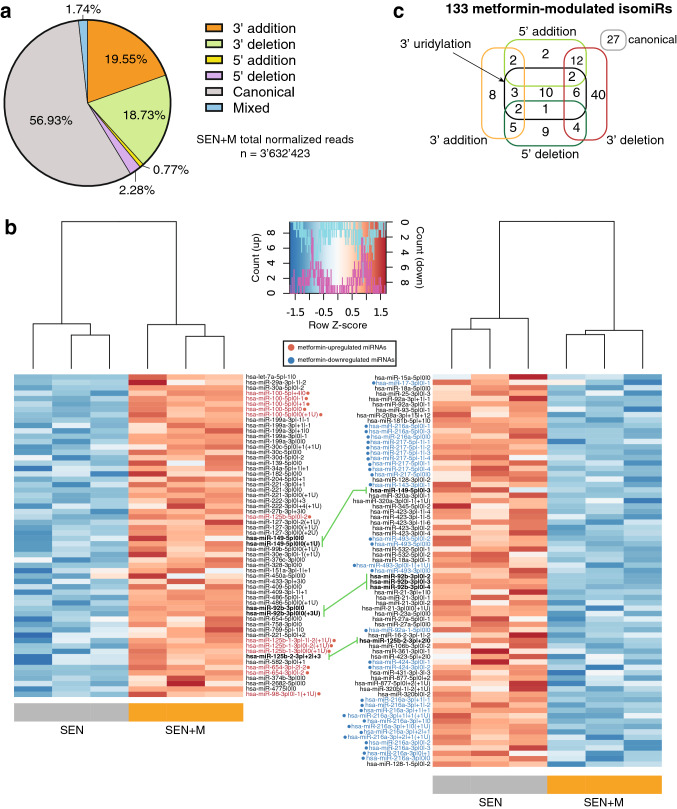
IsomiR analysis of senescent HUVECs treated with metformin. (**a**) Pie chart showing the proportion of isomiR variations in SEN + M samples. (**b**) Heatmaps showing clustering of samples, and isomiRs upregulated (left panel) and downregulated (right panel) in SEN + M compared to SEN, with a FC ≥ 1.5 and FDR < 0.05 cut-off. Data is shown following Z-score transformation. Red color indicates Z-scores > 0 (above mean), blue color indicates Z-scores < 0 (below mean). IsomiR labels are marked and colored in red or blue according to the upregulation or downregulation of their parent miRNA in SEN + M, respectively. Green lines connect variations of the same miRNA (labeled in bold) showing opposite modulation between SEN + M and SEN. IsomiRs are ordered by the MIMAT ID of the parent miRNA. For the isomiR nomenclature, the reader is referred to the Materials and Methods section. (**c**) Diagram reporting the frequencies of the different type of variations among isomiRs modulated by metformin treatment.

Figure 3a shows the contribution of different sequence isoforms to the total miRNA pool in SEN + M. On a total of 3,632,423 reads, the 43.1% was mapped to non-canonical isoforms. No statistically significant difference in the proportion of isomiR variations between SEN and SEN + M was observed (p = 0.103).

The heatmap showed that 133 isomiRs, which are variants of a total of 73 individual miRNAs, were significantly deregulated in SEN + M vs SEN (Fig. 3b). Specifically, 43 isomiRs were isoforms of 14 miRNAs significantly deregulated by metformin treatment (miR-17-3p, -100-5p, -216a-3p, -216a-5p, -217-5p, -125b-5p, -143-3p, -493-3p, -493-5p, -92a-1-5p, -125b-1-3p, -424-3p, -654-3p, -98-3p) (Fig. 3b, red and blue highlights refer to up-/down-regulated miRNAs, respectively).

Among the remaining 90 deregulated isomiRs (related to a total of 59 miRNAs not significantly modulated by metformin), 48 were up-regulated and 42 were down-regulated in SEN + M cells (Fig. 3b, in black). These 59 miRNAs, though not modulated by the treatment as a whole group, encompass at least one isomiR that is differentially regulated by metformin. Notably, 3 miRNAs which were not differentially regulated between SEN + M and SEN, i.e. miR-92b-3p, -149-5p and -125b-2-3p, included isomiRs showing opposite regulations across the two different conditions. In addition, metformin induced the downregulation of isomiRs from 3 members of the miR-17/92 cluster, i.e. miR-17-3p, miR-18a-5p, and miR-92a-3p (Fig. 3b, right panel).

Of note, 39 of 133 metformin-modulated isomiRs showed a modification at the 5′ end (Fig. 3c), which leads to a shift of the seed sequence resulting in a change of the miRNA-target binding site^26^. Moreover, in some instances the seed sequence of the 5′ isomiR is identical to the seed sequence of another canonical microRNA. Indeed, miR-27b-3p|+ 3|0, miR-29a-3p|-1|-2, miR-34a-5p|+ 1|+ 1 and miR-423-5p|+ 2|0 share the same seed sequence of miR-5693, miR-5682, miR-6499-3p and miR-486-3p, respectively. Among the differentially regulated isomiRs, the most frequent modification was the 3′ deletion. Furthermore, 3′uridylation was extensively represented among all isomiR types, except those presenting a 5′ nucleotide addition (Fig. 3c). Interestingly, metformin affected the expression of 24 3′-uridylated miRNAs (Fig. 3b,c).

**Figure 4:**
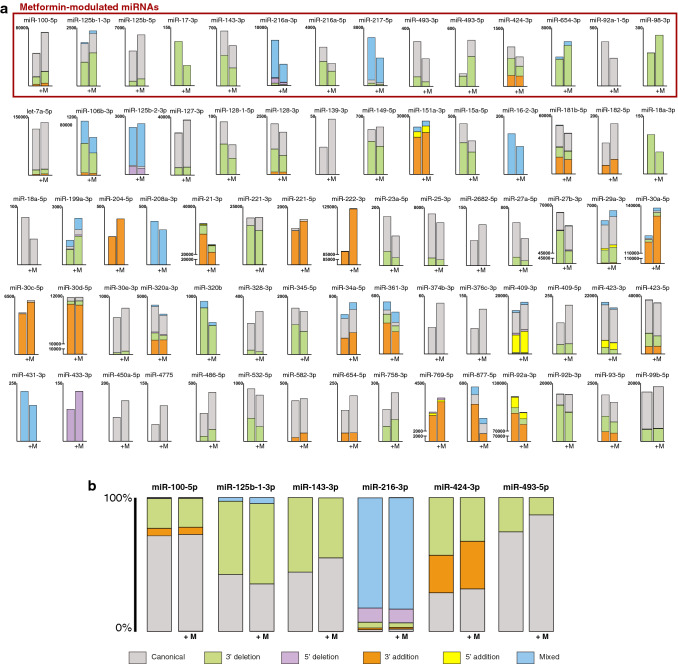
Proportions of isomiR variations within miRNAs modulated by metformin. (**a**) Comparison of normalized reads of the different types of isomiR variations of miRNAs including at least one isomiR differentially regulated by metformin. The 14 metformin-modulated miRNAs including at least one differentially regulated isomiR are highlighted. (**b**) Proportions of isomiR variation types within 6 out of the 27 miRNAs differentially regulated by metformin showing significant isomiR redistribution between SEN and SEN + M.

Figure 4a shows the expression of the different isomiR variants of the 73 miRNAs including at least one isomiR differentially regulated by metformin. Notably, we observed a high variability in the proportion of isomiR variants among the evaluated miRNAs. Indeed, the canonical form, i.e. the one reported in the miRBase database, is not always prevalent (e.g. in the miR-30 family) and some miRNAs included non-canonical variants.

Moreover, metformin induced a significant redistribution of the isomiR variant proportions within 6 out of the 27 differentially regulated miRNAs (Fig. 4b). Only one isoform was detected for 3 miRNAs (miR-17-3p, miR-98-3p, and miR-92–1-5p), whereas no isoforms were detected for the remaining 12 miRNAs. The proportions of the different isomiR variation types between SEN and SEN + M are reported in Table 1.

**Table 1:** Proportions (expressed as %) of isomiR variations among 15 miRNAs differentially regulated by metformin with at least one detected isomiR.

microRNA	Condition	Canonical	Templated modifications				NTA
			3′ addition	3′ deletion	5′ deletion	Mixed	3′ uridylation
miR-17-3p	Sen			100			
	Sen + M			100			
	p-value			–			
miR-100-5p	Sen	71.27	5.63	22.21	0.63	0.26	0.81
	Sen + M	72.21	5.34	21.66	0.56	0.23	1.00
	p-value	0.007	0.033	0.026	0.126	0.308	0.001
miR-216a-5p	Sen	37.40		62.60			
	Sen + M	35.33		64.67			
	p-value	0.180		0.180			
miR-217-5p	Sen	6.80		5.33		87.87	7.16
	Sen + M	5.91		5.34		88.75	8.40
	p-value	0.075		0.984		0.180	0.021
miR-125b-5p	Sen	86.16		13.84			
	Sen + M	85.87		14.13			
	p-value	0.674		0.674			
miR-143-3p	Sen	44.29		55.71			
	Sen + M	55.05		44.95			
	p-value	< 0.001		< 0.001			
miR-493-5p	Sen	74.32		25.68			
	Sen + M	86.96		13.04			
	p-value	< 0.001		< 0.001			
miR-493-3p	Sen	78.20		21.80			21.80
	Sen + M	82.90		17.10			17.10
	p-value	0.201		0.201			0.201
miR-92a-1-5p	Sen	100					
	Sen + M	100					
	p-value	–					
miR-125b-1-3p	Sen	42.42		54.81		2.78	40.64
	Sen + M	35.38		60.08		4.54	50.11
	p-value	< 0.001		0.001		0.003	< 0.001
miR-193a-5p	Sen	43.38		56.62			11.79
	Sen + M	40.68		59.32			10.23
	p-value	0.596		0.596			0.638
miR-424-3p	Sen	28.77	27.99	43.23			
	Sen + M	31.70	35.53	32.77			
	p-value	0.180	0.001	< 0.001			
miR-654-3p	Sen			91.95		8.05	5.25
	Sen + M			91.29		8.71	4.45
	p-value			0.242		0.242	0.064
miR-98-3p	Sen			100			100
	Sen + M			100			100
	p-value			–			–
miR-216a-3p	Sen	1.44	1.27	4.32	10.70	82.27	9.46
	Sen + M	1.75	1.45	3.32	10.26	83.22	9.58
	p-value	0.208	0.430	0.010	0.472	0.208	0.834

*M* metformin, *NTA* non-template addition, *Sen* senescent HUVECs.

P-values for z test.

### MiRNA/isomiR expression trends in endothelial cells during replicative senescence

**Figure 5:**
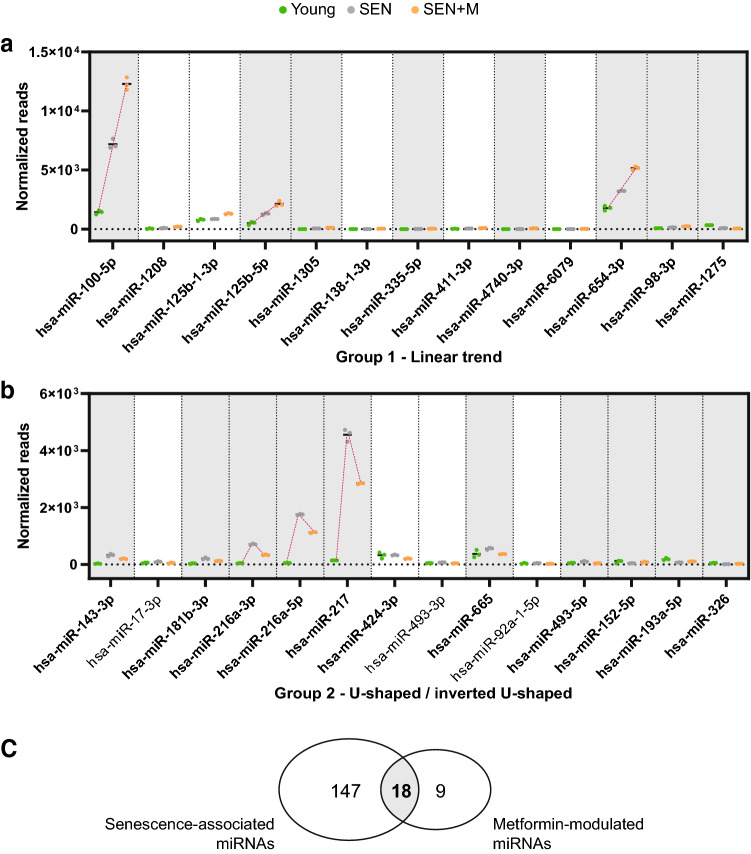
Influence of metformin treatment on senescence-associated miRNA modulation. Normalized reads of 27 miRNAs differentially modulated in Young (green), SEN (gray), and SEN + M (orange), grouped according to the linear (**a**) or U-shaped/inverted U-shaped (**b**) pattern of modulation. MiRNAs with a significant likelihood-ratio test are highlighted in bold. Senescence-associated miRNAs are highlighted with a gray background. (**c**) Venn diagram reporting the number of miRNAs differentially regulated in SEN compared to Young cells and in SEN + M compared to SEN.

To gain insight into the biological significance of miRNA/isomiR modulation induced by metformin treatment in senescent HUVECs (SEN, SEN + M) we used non-senescent HUVECs as control (Young, SA β-gal < 5%, Fig. 1b)^22^. This strategy allowed us to identify two different trends in miRNA modulation and to separate the 27 miRNAs according to their pattern of modulation. On one hand, 13 miRNAs were characterized by linear increasing or decreasing trend, when the Young/SEN/SEN + M sequence was examined (Fig. 5a, group 1). The most evident linear trends were observed for miR-100, -125b-5p, and -654-3p, showing the most abundant expression. Metformin treatment further increases the expression of these miRNAs in senescent cells. On the other hand, 14 miRNAs were characterized by a ‘U-shaped’/ ‘inverted U-shaped’ trend of the Young/SEN/SEN + M sequence, suggesting that metformin induced a (partial) reversal of the miRNA expression induced by senescence (Fig. 5b, group 2). Among the 14 miRNAs belonging to this latter group, the ‘U-shaped’/ ‘inverted U-shaped’ trend was confirmed by a significant likelihood ratio test (adjusted p value < 0.05) (in bold in Fig. 5b). The most relevant inverted U-shaped trends were observed for miR-217-5p, -216a-3p, and -216a-5p. Overall, metformin affected the expression of 18 SA miRNAs (Fig. 5c) and notably was able to rescue the miR-216a and miR-217-5p overexpression in senescent cells previously reported by our group^22^.

A similar approach was carried out on the 133 differentially regulated isomiRs. Results of the likelihood ratio test performed on Young, SEN, and SEN + M samples revealed significant U-shaped/inverted U-shaped trends for 75 isomiRs and linear trends for 49 isomiRs, belonging to 52 and 20 individual miRNAs, respectively. Notably, 5 miRNAs, i.e. miR-92b-3p, -149-5p, -221-3p, -222-3p, 532-5p, included isomiRs following either U-shaped or linear trends (data not shown).

### Metformin alters the miRNA and isomiR targetome of senescent endothelial cells

**Figure 6:**
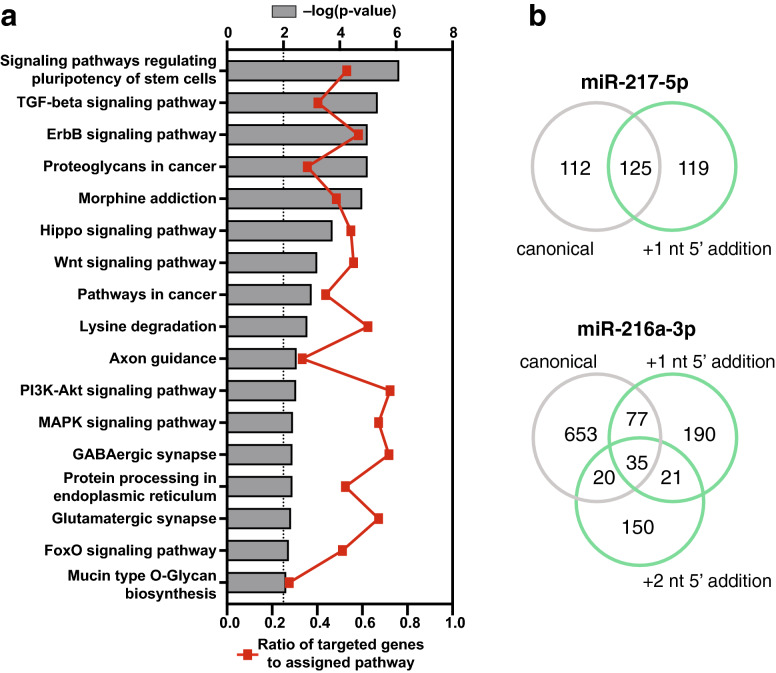
Target analysis of miRNAs and isomiRs affected by metformin. (**a**) KEGG pathways significantly enriched in predicted target genes of the 11 miRNAs showing a significant U-shaped/inverted U-shaped trend among Young/SEN/SEN + M samples. Pathways are ranked according to the significance of enrichment (grey bars, upper y-axis). Ratios referring to the proportion of targeted genes related to the total number of genes in each pathway are displayed (red line graph, bottom y-axis). (**b**) Results of the TargetScan custom analysis on the canonical and 5′ isomiR seed sequences of miR-217-5p and miR-216-3p.

To explore target genes and pathways affected by the 11 miRNAs showing significant U-shaped or inverted U-shaped trends, pathway enrichment analysis was performed using the miRPath v.3/Diana tool. Figure 6a lists the involved KEGG pathways (p < 0.01) ranked by the significance of the enrichment. The proportion of targeted genes over total genes for each pathway is also reported. A considerable number of pathways related to cell proliferation, i.e. TGFβ, ErbB, Wnt and MAPK pathways, is significantly enriched. Notably, the PI3K-Akt-mTOR pathway is the one containing the greatest amount of targeted genes (72.3%), in agreement with the inhibitory effects of metformin on mTOR signaling^27^.

Among these miRNAs, we focused on the only two miRNAs showing detectable levels of at least one 5′ isoform, i.e. miR-217-5p and miR-216a-3p. Interestingly, the canonical/3′ and the 5′ miR-217-5p isomiRs share only half of the predicted targets, while the other half is exclusive to either seed sequence. Regarding miR-216a-3p, the deletion of one or two nucleotides at the 5′ end leads to the generation of two alternative seed sequences. The 3 different seed sequences shared only a small pool (35) of predicted targets (Fig. 6b). The target genes of canonical and 5′isomiR seed sequences were evaluated also for those miRNAs including at least one 5′isomiR presenting a significant linear or U-shaped trend in Young, SEN, and SEN + M (Supplementary Figure S2). Notably, the canonical form and the 5′ isomiR of miR-100-5p shared no predicted target genes.

## Discussion

**Figure 7:**
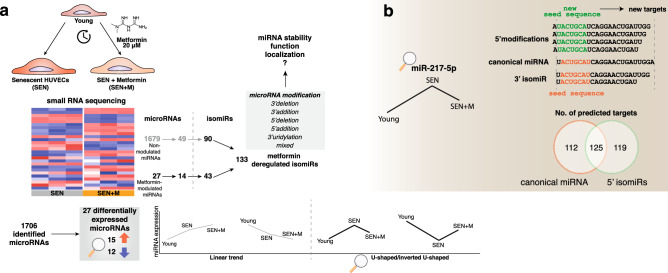
(**a**) Summary of the effects of metformin treatment on the miRNA/isomiR pool of HUVECs undergoing replicative senescence. Metformin differentially regulates the expression of 27 miRNAs. Two different trends in miRNA modulation were observed with reference to the Young/SEN/SEN + M sequence, i.e. a linear increasing/decreasing trend and a ‘U-shaped’/‘inverted U-shaped’ trend. Moreover, metformin treatment altered the expression of 133 isomiRs, related to 14 differentially expressed miRNAs and 59 non differentially expressed miRNAs. (**b**) Metformin treatment induced a partial reversal of the senescence-associated expression of miR-217-5p, including its 5′ isomiRs, which are associated to a shifting of the seed sequence. The inclusion of these additional seed sequences into the targetome analysis yielded a considerably greater number of target genes, most of which were not shared with the canonical miRNAs.

In the present study, we investigated for the first time the miRNA landscape in endothelial cells (ECs) undergoing replicative senescence after a long-term treatment with metformin. Surprisingly, only 27 miRNAs on a total of 1706 detected by the small RNA-seq analysis were differentially regulated by metformin, despite the long duration of the exposure to a pharmacologically pertinent dose of the drug. To gain insight into the biological significance of these modulations, we used young proliferating HUVECs as reference group, in order to identify specific trends of modulation. We focused on the group of miRNAs characterized by a U-shaped/inverted U-shaped trend of expression in young vs. SEN vs. SEN + M, since this peculiar trend could reflect the ability of metformin to modulate the trajectories of senescence associated miRNAs (Fig. 7a). Increasing evidence suggests that a number of biomarkers of human aging followed non-linear trends when subjects representing the extreme phenotype of successful aging, i.e. the centenarians, are included in the analysis^28–32^. We therefore employed an in vitro cellular senescence model mimicking the gradual deterioration of endothelial function that accompanies human aging^33^, to unravel the ability of metformin to affect the senescence-associated miRNA/isomiR modulation.

This approach allowed us to show that metformin can revert the SA trend of a number of miRNAs that were extensively studied in the context of cellular aging, including miR-216-3p, -216-5p, and -217-5p, which we previously identified among the most upregulated miRNAs in senescent HUVECs^22^. MiR-217-5p was proved to be involved in EC and human fibroblast senescence by targeting SIRT1 and DNMT1, respectively^34,35^. Furthermore, we recently demonstrated that the same pro-senescence effects of miR-217 can be spread through the exchange of small extracellular vesicles^22^. Similarly, miR-216a was shown to be involved in EC aging, in atherosclerosis-related endothelial dysfunction by impairing the autophagy response to the accumulation of oxidized low-density lipoproteins^36^, and in macrophage pro-inflammatory M1 polarization by boosting the NF-κB pathway^37,38^. Among miRNAs showing a linear trend in Young vs. SEN vs. SEN + M, miR-100-5p was previously shown to be upregulated in senescent HUVECs^22,39^, while the metformin-mediated induction of miR-125-5p was consistent with previous reports on macrophages^40^ and senescent ECs^12^.

Regarding the analysis of isomiRs, this is the first deep sequencing assessment of isomiRs in senescent HUVECs. One miRNA gene can potentially produce multiple distinct isomiRs, differing in length, sequence, or both^26^. Our results proved that the assessment of isomiRs can unravel complex modulations of the miRNA pool not detectable with standard miRNA analysis. Indeed, isomiR analysis allowed us to fully uncover the downregulating effects of metformin on the miR-17/92 cluster, which has been previously shown to be over-represented in a wide range of cancers and cardiovascular diseases and downregulated in physiological aging^41^. Therefore, further developments of isomiR analysis are warranted to increase our knowledge on miRNA modulation in a number of physiological and pathological processes.

In agreement with previous reports^25,42^, we observed a considerable presence of 3′ isomiRs, while more than half of the total reads was mapped to canonical miRNAs. It has to be noted, however, that the term ‘canonical’ refers to the sequence annotated in miRBase and do not necessarily indicate the most abundant miRNA isoform in a specific cell type or tissue or the primary product of pre-miRNA cleavage^43^.

On the other hand, only a small number of reads (about 3%) mapped to 5′ isomiRs, which are associated to a shifting of the seed sequence (Fig. 7b). For this reason, we evaluated the number of targets shared by the isoforms of miR-216a-3p and miR-217-5p, which were both modulated by metformin treatment and expressed 5′ isoforms. The inclusion of these additional seed sequences into the targetome analysis yielded a considerably greater number of target genes, most of which were not shared with the canonical miRNAs. As expected, the coexistence of more than one 5′ isomiR, as in the case of miR-216-3p, proportionally increased the amount of target genes. The ability of isomiRs of being loaded onto the RISC complex support their possible biological role^19,44^. Indeed, a previous report showed that the ratio between miR-411 and its 5′ isomiR in ECs is affected by acute ischemia and that only the 5′ isoform of miR-411 is capable of impairing angiogenesis by targeting a different subset of mRNAs^19^.

The 3′ end miRNA modifications are mostly related to post transcriptional deletion of nucleotides, i.e. trimming, or the addition of one or more nucleotides, i.e. tailing^45^. It has to be noted, however, that is quite challenging to distinguish templated nucleotides added during miRNA maturation from those added post-transcriptionally to the mature miRNA. In our study, we assessed isomiRs resulting from the untemplated nucleotide addition to the 3′ end of pre-miRNA or mature miRNA^46^. While these modifications are not associated with a shifting of the seed sequence, it has been demonstrated that 3′ uridylation enhances base-pairing between tailed miRNA and targets, a phenomenon named as tail-U-mediated repression (TUMR). Therefore, TUMR expands the miRNA target repertoires by producing novel miRNA-target binding sites in the presence of an incomplete seed-pairing^18^. Moreover, 3′ post-transcriptional modifications were shown to affect miRNA stability^47^, intracellular levels, and compartmentalization into extracellular vesicles^20^. Notably, miRNAs are not the sole substrates of the 3′ uridylation mediated by terminal uridyltransferases (TUTs). Indeed, 3ʹ-terminal uridylation of viral RNAs in mammalian cells has been recently identified as a conserved antiviral defense mechanism^48^. Interestingly, metformin affected the expression of 22 3′-uridylated miRNAs; therefore, it is straightforward to conceive a framework in which metformin could impact cellular senescence through the modulation of miRNA function, stability, and localization.

Our in vitro results support the role of isomiR assessment in biological samples as a useful tool to improve our knowledge on the aging process or discover new biomarkers of biological aging.

Nevertheless, several limitations need to be acknowledged. The study design does not allow to draw any mechanistical conclusion on the role of metformin on ECs or cellular senescence. In addition, some of the mechanisms of isomiR biogenesis are still unclear, implying the intrinsic difficulty to assess whether 3′ nucleotide addition occurs during or after miRNA transcription. Finally, qPCR validation of NGS assessment of isomiRs is still hampered by analytical challenges, such as the absence of dedicated protocols and reagents, e.g. probes and primers, and the relative inefficiency of the currently available techniques in differentiating highly similar sequences^49,50^.

NGS studies on isomiRs paved the way to the exploration of novel non-canonical targets and allowed the identification of new regulatory mechanisms of miRNA expression and intracellular localization, adding an additional layer of complexity to the study of the epigenetic variations accompanying cell senescence, although further investigations are required to better understand the biological functions of the cellular isomiR pool.

Overall, we showed that long-term treatment with metformin is able to partly attenuate the complex miRNA/isomiR remodeling observed during cellular senescence in ECs, supporting further exploration of the impact of metformin on the cellular epigenetic landscape as a possible mediator of the putative beneficial effect of this drug on the aging process.

## Materials and methods

### Cell culture and treatment

An in vitro model of endothelial replicative cell senescence was established using long-term cultured HUVECs. Cryopreserved HUVECs obtained from pool of donors were purchased from Clonetics (Lonza, Switzerland) and cultured in EGM-2 (CC-3162, Lonza) at 37 °C in a humidified atmosphere containing 5% CO_2_. Cells were seeded at a density of 5000/cm^2^ and sub-cultured when they reached 70–80% confluence. All cells tested negative for mycoplasma infection. Before replating, harvested cells were counted using a hemocytometer. Population doublings (PDs) were calculated by the formula: (log_10_F – log_10_I)/log_10_2, where F is the number of cells at the end of the passage and I is the number of seeded cells. Cumulative population doubling (cPD) was calculated as the sum of PD changes. Cells were cultured until the arrest of replication and classified based on SA β-galactosidase (β-gal) activity into young (SA β-gal < 5%) and senescent (SEN, SA β-gal > 80%) cells using Senescence Detection Kit (cat. no. K320, BioVision Inc., USA) as described previously^21^. Cells were treated with 20 μM metformin (cat. D150959, Sigma Aldrich, Italy) added at each medium replacement.

### RNA extraction

Total RNA, including small (< 200 nucleotides) RNAs, was extracted from HUVEC pellets using Norgen total RNA Purification Kit (cat. no. 37500, Norgen Biotek Corporation, Canada) according to the manufacturer’s protocol. Purified RNA was stored at − 80 °C until analysis.

### mRNA expression level

*CDKN2A* mRNA expression was assessed as previously described^22^. Primer sequences (written 5′-3′) were as follows: p16, Fw: CATAGATGCCGCGGAAGGT, Rv: CTAAGTTTCCCGAGGTTTCTCAGA; β-actin, Fw:TGCTATCCCTGTACGCCTCT, Rv: GTGGTGGTGAAGCTGTAGCC. Primer concentration was 200 nM. Delta delta Ct method was performed to analyze the results and Young cells were used as reference group.

### Small RNA sequencing analysis

Small RNA sequencing was performed in triplicate on Young and SEN cells, and SEN cells treated with metformin. TruSeq Small RNA Library PrepKit v2 (Illumina; RS-200-0012/24/36/48) was used for library preparation according to the manufacturer’s indications. Briefly, 35 ng purified RNA was linked to RNA 3′ and 5′ adapters, converted to cDNA, and amplified using Illumina primers containing unique indexes for each sample. Each library was quantified using Agilent Bioanalyzer and High Sensitivity DNA Kit (cat. no. 5067-4626, Agilent Technologies, USA) and equal amounts of libraries were pooled together. Size selection allowed keeping 130–160 bp fragments. After ethanol precipitation, the library pool was quantified with Agilent High Sensitivity DNA Kit, diluted to 1.8 pM, and sequenced using NextSeq 500/550 High Output Kit v2 (75 cycles) (Illumina; FC-404-2005) on the Illumina NextSeq500 platform.

Raw base-call data generated by the Illumina NextSeq 500 system were demultiplexed using Illumina BaseSpace Sequence Hub (https://basespace.illumina.com/home/index) and converted to FASTQ format. After a quality check with FastQC (https://www.bioinformatics.babraham.ac.uk/projects/fastqc/), sequence reads were quality trimmed using the cutadapt tool^51^. Sequence reads were aligned to the miRBase version 21.0 database^52^ using the STAR algorithm^53^. Standard miRNA quantification (including the canonical form and all isoforms) was obtained as previously detailed^22^.

### Quantification of miRNA isoforms

Sequence reads were quality trimmed using the cutadapt tool, and mapped unambiguously using SHRIMP2^54^ to the human genome assembly GRCh38. During the mapping, no insertions or deletions, and at most one mismatch was permitted. IsomiRs were identified as done previously^16,17,55–57^. The isomiR nomenclature used is based upon the one used previously in Loher et al.^17^. For example, the isomiR whose 5′ terminus begins one position to the right (+ 1) of the archetype’s 5′ terminus and whose 3′ terminus ends two positions to the left (− 2) of the archetype’s 3′ terminus is labeled “ + 1|− 2”. The archetype isomiR, the sequence found in public databases, is labeled as “0|0”.

IsomiR abundances were quantified in reads per million (RPM). Only reads that passed quality trimming and filtering and could be aligned exactly to miRNA arms were used in the denominator of this calculation. The abundance of a miRNA arm is calculated as the sum of normalized abundances of all isomiRs from the arm.

Raw and processed datasets have been deposited in NCBI’s Gene Expression Omnibus (GEO) (https://www.ncbi.nlm.nih.gov/geo) with accession reference GSE149771.

### Statistical analysis of small RNA-seq data

Data analysis was carried out using the DESeq2 1.26.0^58^ Bioconductor package within the R version 3.6.1 environment. MiRNAs/isomiRs showing a differential expression between SEN and SEN + M were identified using a fold change ≥ 1.5 filter and an FDR < 5% cut-off at two-tailed moderated *t*-test with Benjamini–Hochberg correction. A two-tailed likelihood ratio test (LRT) was used to compare miRNA/isomiR expression among Young, SEN, and SEN + M samples, with a Benjamini–Hochberg FDR < 5%. The significance of the differences between isomiR proportions within each miRNA was tested using z-test. The PCA plot and correlation matrix showing Pearson’s correlations among samples were created using the pcaExplorer version 2.12.0 R/Bioconductor package^59^. Heatmaps were produced using the heatmap2 function from the R package gplots version 3.0.3 (https://cran.r-project.org/web/packages/gplots/) with row scaling and hierarchical clustering of the rLog transformed expression values.

### MiRNA target prediction

Putative miRNA targets were individuated using the Diana mirPath v.3 platform and the tools TarBase v7.0 and microT-CDS v5.0, which allow the analysis of KEGG pathways enrichment^60,61^ for experimentally validated and predicted target genes, respectively^62^. The analysis was carried out using the ‘pathways union’ option. P-values were calculated by the Fisher’s exact test and the false discovery rate (FDR) was estimated using the Benjamini and Hochberg method. A p-value threshold of 0.01 was applied. Differential target genes of the canonical/3′ isomiRs and 5′ isomiRs were predicted using the TargetScan Custom tool v. 5.2 (http://www.targetscan.org/vert_50/seedmatch.html), which searches for a complementary 3′ UTR against a provided seed sequence.

## Supplementary Information

Supplementary Information

## Data Availability

Raw and processed datasets have been deposited in NCBI’s Gene Expression Omnibus (GEO) (https://www.ncbi.nlm.nih.gov/geo) with accession reference GSE149771.
