# Supplementary material for: Long-term exposure of human endothelial cells to metformin modulates miRNAs and isomiRs
Source: Sci Rep. 2020 Dec 11;10:21782. doi: 10.1038/s41598-020-78871-5 (PMC7732983; doi:10.1038/s41598-020-78871-5)

Long-term exposure of human endothelial cells to metformin modulates miRNAs and isomiRs

Angelica Giuliani*, Eric Londin*, Manuela Ferracin, Emanuela Mensà, Francesco Prattichizzo, Deborah Ramini, Fiorella Marcheselli, Rina Recchioni, Maria Rita Rippo, Massimiliano Bonafè, Isidore Rigoutsos, Fabiola Olivieri, Jacopo Sabbatinelli

SUPPLEMENTARY FIGURES

Supplementary Figure S1. Pairwise sample-to-sample scatter plots of normalized counts from miR-seq analysis of n=3 SEN and n=3 SEN+M samples.

Supplementary Figure S2. Number of predicted target genes of canonical (gray) and 5' isomiRs (green) of miRNAs including at least one 5'isomiR showing a significant linear (group 1) or U-shaped/inverted U-shaped (group 2) trend in Young, SEN, and SEN+M samples.

Supplementary Figure S1. Pairwise sample-to-sample scatter plots of normalized counts from miRseq analysis of n=3 SEN and n=3 SEN+M samples.

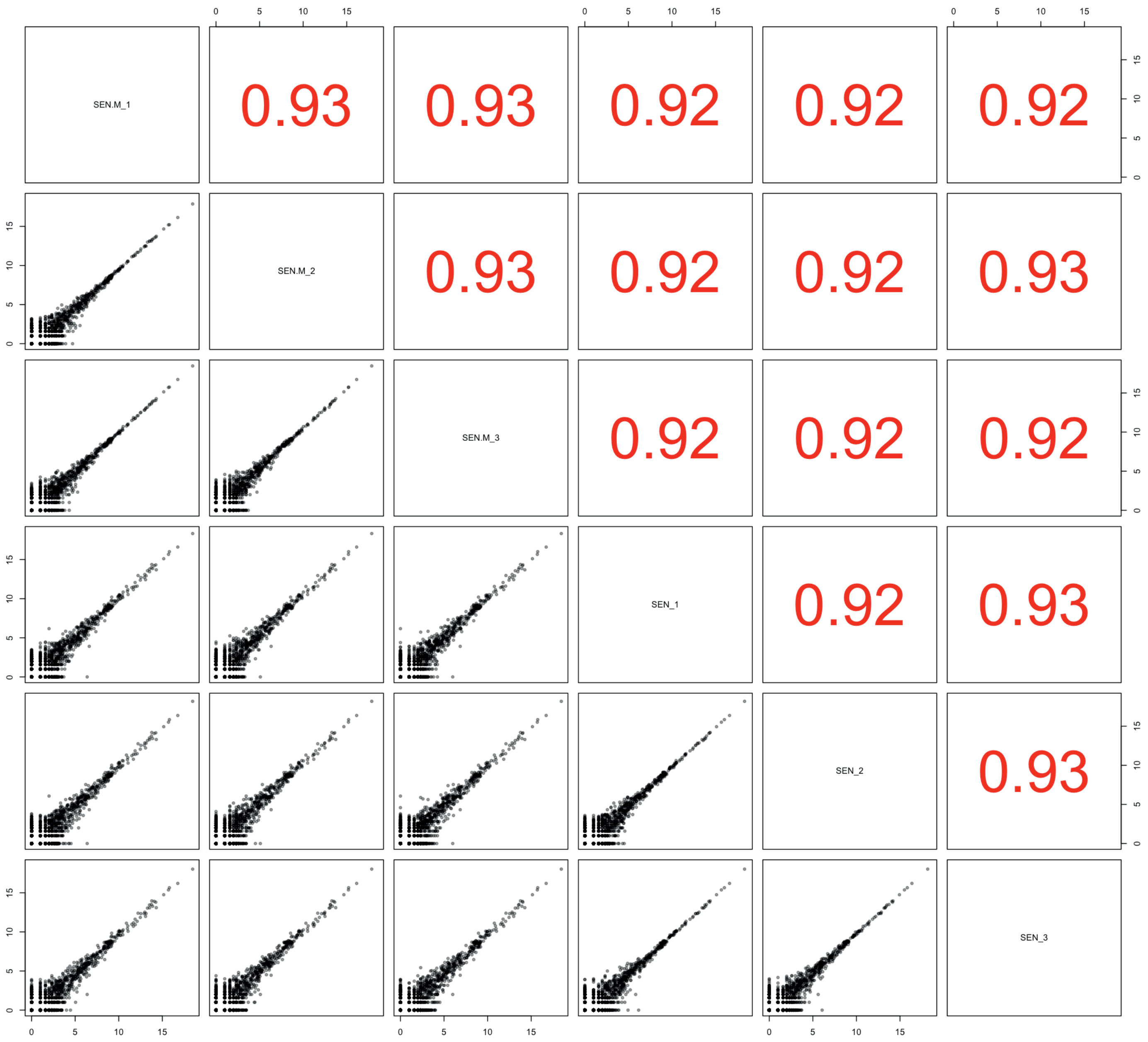

Supplementary Figure S2. Number of predicted target genes of canonical (gray) and 5' isomiRs (green) of miRNAs including at least one 5'isomiR showing a significant linear (group 1) or Ushaped/ inverted U-shaped (group 2) trend in Young, SEN, and SEN+M samples.

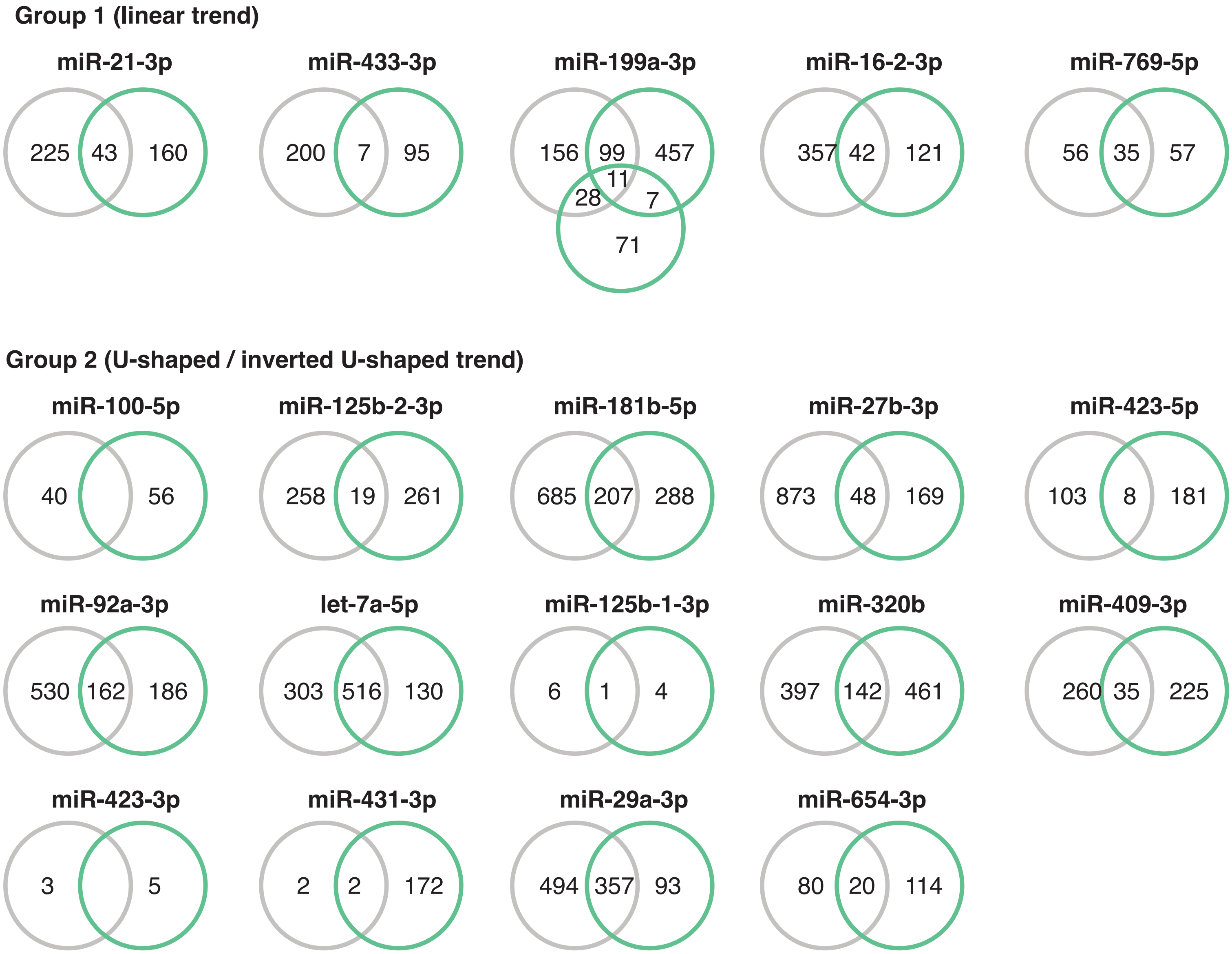

Supplement: Supplementary file 1 — Supplementary Information [file 41598_2020_78871_MOESM1_ESM.pdf]
